# Association of Medicaid Financing and Concentration of Assisted Living Residents Dually Eligible for Medicare and Medicaid

**DOI:** 10.1001/jamahealthforum.2022.5338

**Published:** 2023-02-03

**Authors:** Portia Y. Cornell, Cassandra Hua, Momotazur Rahman, Gauri Gadkari, Rachel Gunderson, Lindsey Smith, Kali S. Thomas

**Affiliations:** 1 Department of Health Services, Policy, and Practice, Brown University, Providence, Rhode Island; 2 Center of Innovation for Long-Term Services and Supports, Providence Veterans Administration Medical Center, Providence, Rhode Island; 3 Department of Economics, Brown University, Providence, Rhode Island

## Abstract

This cross-sectional study examines different levels of state Medicaid financing for assisted living and the association with the number of beneficiaries with dual Medicare and Medicaid eligibility who live in assisted living facilities.

## Introduction

Almost 1 in 5 assisted living (AL) residents rely on Medicaid to pay for personal care and supportive services.^1,2^ High concentrations of residents dually eligible for Medicare and Medicaid in nursing homes are associated with lower staffing levels and more state-identified deficiencies.^3,4^ This study assessed the concentration of individuals dually eligible for Medicare and Medicaid in AL communities and the association with Medicaid financing of AL services.

## Methods

In this cross-sectional study, we used 2018 Medicare enrollment data, a national directory of licensed AL communities, and records of Medicaid state waivers and state plans with coverage for AL in 47 states. Data were analyzed January to November 2022. We identified beneficiaries residing in large (≥25 beds) AL communities using 9-digit zip codes.^5^ Additional information is presented in the eMethods in Supplement 1. The Brown University Institutional Review Board approved the study and waived the informed consent requirement because we used secondary administrative data. We followed the STROBE reporting guideline.

We calculated the Gini coefficient, a measure of states’ residential concentration of adults dually eligible for Medicare and Medicaid within AL communities, as our main outcome measure and within zip codes as a proxy for geographic distribution. A Gini coefficient of 0 represents even distribution of these residents across every AL community and zip code, and 1 reflects maximum concentration (all concentrated in 1 AL community and zip code). We categorized states by whether they had a Medicaid waiver or a state plan amendment that covered AL services in 2019. We compared the state Gini coefficient across these categories using ordinary least-squares regression. A 2-sided *P* < .05 was considered statistically significant.

## Results

We identified 57 526 474 adults enrolled in Medicare in 2018. In AL, we identified 471 695 adults across 12 168 AL communities, of whom 116 758 (25%) were dually enrolled in Medicaid. In non-AL settings, we identified 57 054 779 adults across 38 667 zip codes, of whom 11 011 817 (19%) were dually enrolled.

Nationally, 78% of dually eligible individuals resided in 20% of AL communities. The distribution of these adults was similar between AL communities and zip codes (Gini coefficient, 0.74). By state, Gini coefficients for dually eligible adults in AL communities ranged from 0.52 in Washington (lowest concentration) to 0.88 in Alabama (highest concentration) (Figure). Most states covered services in AL communities with a Medicaid waiver; 5 states had no coverage. States with no Medicaid financing for AL had the highest Gini coefficient (median, 0.80 [IQR, 0.78-0.84]), followed by waiver only (median, 0.70 [IQR, 0.67-0.79]). States that covered AL through a state plan (median, 0.62 [IQR, 0.61-0.70]) or both a state plan and waiver (median, 0.64 [IQR, 0.55-0.73]) had the lowest Gini coefficients (Table). In a regression model, states that covered AL services with a Medicaid state plan, waiver, or both had significantly lower median Gini coefficients than states with no coverage (Table). The zip code Gini coefficient for people dually eligible for Medicare and Medicaid outside of AL communities was not statistically significant and did not substantially change other estimates in the model.

**Figure.**
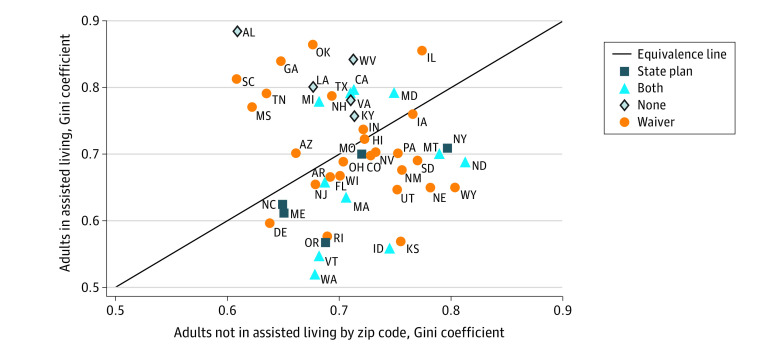
Scatter Diagram of States’ Concentration of Dually Eligible Residents in Assisted Living vs Dually Eligible Individuals in the Community The Gini coefficient of the distribution of dually eligible residents of assisted living communities vs the Gini coefficient of dually eligible individuals not in assisted living by zip code are shown. A higher Gini coefficient indicates greater concentration. The diagonal line represents equivalent assisted living and zip code Gini coefficients. Alaska, Minnesota, and Connecticut are not included.

**Table.** Differences in Gini Coefficient Associated With Medicaid Support for Assisted Living (AL) vs States With No Waiver or State Plan^a^

Medicaid funding for AL	No. of states	Gini coefficient, median (IQR)	Regression of Gini coefficients on AL Medicaid type			
			Coefficients (95% CI)	*P* value	Coefficients (95% CI)	*P* value
No Medicaid payment for services in AL	5	0.80 (0.78 to 0.84)	0.81 (0.74 to 0.88)^b^	<.001	NR	NR
State plan amendment^c^	5	0.62 (0.61 to 0.70)	−0.17 (−0.27 to −0.07)	.002	−0.17 (0.28 to −0.06)	.003
Waiver only^d^	29	0.70 (0.67 to 0.79)	−0.10 (−0.18 to −0.02)	.02	−0.10 (−0.18 to −0.06)	.02
Both state plan amendment and waiver	8	0.64 (0.55 to 0.73)	−0.17 (−0.26 to −0.08)	<.001	−0.11 (−0.23 to −0.01)	.06
Geographic concentration (zip code Gini)^e^	NA	0.71 (0.68 to 0.75)	NR	NA	−0.33 (−0.85 to −0.19)	.21

Abbreviations: NA, not applicable; NR, not reported.

^a^ Data on 47 states (excluding Alaska, Minnesota, and Connecticut). Outcome of regression models is the state-level Gini coefficient representing concentration of dually eligible residents among AL communities.

^b^ Intercept.

^c^ Including 1915(i), 1915(j), and 1915(k).

^d^ Including 1915(c) or 1115.

^e^ Geographic concentration is measured using zip code Gini coefficient of the concentration of dually eligible adults among zip codes.

## Discussion

Individuals dually eligible for Medicare and Medicaid are highly concentrated in AL communities, with the lowest concentration among states with state plan amendments to cover AL services and the highest concentration in states with no Medicaid coverage for AL.

This study was limited by the state-level analysis, as either waivers or state plans can be restricted to certain license types within a state. We did not have information about state programs that supplement Medicaid to cover room and board for dually eligible adults. We could not examine distribution across smaller AL settings, which house many dually eligible residents.

Higher concentration of AL residents dually eligible for Medicare and Medicaid could be a disadvantage because Medicaid reimbursement rates are generally lower than private pay rates. Future research is warranted to evaluate AL care quality by the distribution of dually eligible adults.

## Notes

Supplement 1. **eMethods.** Data Repository
eReferences

Supplement 2.
Data Sharing Statement
