# Supplementary material for: Association of Medicaid Financing and Concentration of Assisted Living Residents Dually Eligible for Medicare and Medicaid
Source: JAMA Health Forum. 2023 Feb 3;4(2):e225338. doi: 10.1001/jamahealthforum.2022.5338 (PMC9898816; doi:10.1001/jamahealthforum.2022.5338)
Supplement: Supplement 1. — eMethods. Data Repository eReferences [file jamahealthforum-e225338-s001.pdf]

Supplementary Online Content

Cornell PY, Hua C, Rahman M, et al. Association of Medicaid financing and concentration of assisted living residents dually eligible for Medicare and Medicaid. *JAMA Health Forum.* 4(2):e225338. doi:10.1001/jamahealthforum.2022.5338

eMethods. Data Repository
eReferences.

This supplementary material has been provided by the authors to give readers additional information about their work.

© 2023 Cornell PY et al. *JAMA Health Forum.*

eMethods. Data repository

Analysis code and data used to create figures in the manuscript can be found at the following link: https://doi.org/10.26300/r3zd-4342

Data Sources

We used a national directory of assisted living (AL) communities in 2019, which we created by collecting information regarding licensed AL /residential care facilities from individual state websites and state agents. The state data contained the address, capacity and license information for each community. Only facilities in the continental United States that were licensed to serve an older population and had a capacity of 25 or more beds were included in this study. Minnesota and Connecticut were excluded from the analysis due to its licensing method in 2019, under which the state licensed the "home care agencies" instead of the residential building where the AL services were provided.

We used the Medicare Master Beneficiary Summary File (MBSF) to identify beneficiaries and classified them as dual if they were enrolled in both Medicaid and Medicare at any point in the past 12 months on December 31st, 2018. A ZIP Code History File (ZHF) was used for obtaining beneficiaries' residential ZIP codes along with the start and end date associated with that residence. We identified possible ZIP codes that correspond to AL communities using Medicare Part B Claims (20%) and the Outcome and Assessment Information Set (OASIS). Residents who were in a nursing home on December 31st of 2018 were identified and excluded using the Minimum Data Set (MDS).

Identifying the cohort

AL residents

We limited the cohort to those living at an AL ZIP code on December 31, 2018. We identified AL residents by identifying Medicare Part B claims in the Carrier File that had a Place of Service code #13 and/or Current Procedural Terminology (CPT) codes 99324-99337, indicating that care

© 2023 Cornell PY et al. JAMA Health Forum.

was delivered in an AL setting. We used the OASIS to identify beneficiaries with a start of care assessment that had a living arrangement code indicating “congregate living situation with around-the-clock assistance.” We linked these data to the ZHF to obtain the beneficiaries 9-digit ZIP codes. Each beneficiary was assigned to a specific AL community by matching the first 7 digits of their residence ZIP code to the AL community ZIP codes available in our 2019 national directory. Using the MDS, we excluded beneficiaries in nursing homes on this date, resulting in a cohort of 474,661 AL residents in 12,168 AL communities.

Community residents

Community residents were defined as all the beneficiaries in the MBSF 2018 file who were not in an AL setting or nursing home on December 31,2018. Using the AL resident cohort we excluded those in AL settings from the MBSF 2018 file. The resulting cohort of 58,911,266community residents was grouped by unique 5 digit ZIP codes (N=40,325) across the 47 states.

Statistical analysis

We measured concentration of duals using the Gini index, a measure of concentration that has previously been used to understand geographic segregation.[1] We calculated the proportion of AL residents who were dually eligible in each AL community and the proportion of residents who were dually eligible in each 5 digit ZIP code. We used these ratios to calculate the Gini index among AL communities and among ZIP codes. The Gini index is one of many ways to describe geographic concentration—just, as analogously, the mean is one of several possible measures of central tendency. Two groups could have the same Gini but not have identical distributions**.**

eReferences

1 Firebaugh G, Acciai F. For blacks in America, the gap in neighborhood poverty has declined faster than segregation. *Proc Natl Acad Sci U S A*. 2016;113(47):13372-13377.

© 2023 Cornell PY et al. JAMA Health Forum.
