# Supplementary material for: Association of Medicaid Financing and Concentration of Assisted Living Residents Dually Eligible for Medicare and Medicaid
Source: JAMA Health Forum. 2023 Feb 3;4(2):e225338. doi: 10.1001/jamahealthforum.2022.5338 (PMC9898816; doi:10.1001/jamahealthforum.2022.5338)
Supplement: Supplement 2. — Data Sharing Statement [file jamahealthforum-e225338-s002.pdf]

Data Sharing Statement

Cornell. Association of Medicaid Financing and Concentration of Assisted Living Residents Dually Eligible for Medicare and Medicaid. *JAMA Health Forum*. Published February 03, 2023. doi:10.1001/jamahealthforum.2022.5338

Data
Data available: No

Additional Information
Explanation for why data not available: Medicaid data contains protected health information and cannot be made public, as per data use agreement with CMS.
